# The Clear Cell Odontogenic Carcinoma: Case Report of a Rare Jaw Malignancy

**DOI:** 10.1007/s12663-025-02590-5

**Published:** 2025-07-11

**Authors:** Vasco Starke, Markus Merkl, Lukas Grössing, Mauro Pau, Wolfgang Zemann

**Affiliations:** Department of Oral and Maxillofacial Surgery, Medical University Graz, Auenbruggerplatz 5, 8036 Graz, Austria

**Keywords:** Odontogenic clear cell carcinoma—CCOC, Clear cell ameloblastoma, Malignant odontogenic tumor, Tumor reconstruction

## Abstract

Clear cell odontogenic carcinoma is a very rare malignant tumor of the jaw. Diagnosis and treatment may be challenging as the tumor grows locally aggressive and infiltrative with possible metastatic spread, while the clinical presentation varies, and the definitive diagnosis can only be confirmed by immunohistopathology. The case of a 25-year-old male patient with clear cell odontogenic carcinoma of the mandible is presented. Therapeutic treatment included primary surgical resection with bilateral removal of cervical lymph nodes followed by a two-stage reconstruction approach. Continuous follow-up over 7.5 years showed no signs of tumor recurrence.

## Introduction

The clear cell odontogenic carcinoma (CCOC), formerly also termed clear cell ameloblastoma, is a very rare malign neoplasm of the jaw. According to the most recent systematic review by Labrador et al., there are currently only 117 reported cases in the English literature [1]. The 5th Edition of the World Health Organization Classification of Head and Neck Tumors from 2022 described the CCOC as a rare low-grade malignancy. [2] The locally aggressive, infiltrative growth and possible spread of cervical, pulmonary, or mediastinal metastases, in combination with nonspecific clinical features, complicates the diagnosis and treatment of affected patients [3, 4]. Considering that the number of successfully treated cases reported in the literature is very limited, the aim of this study was to contribute to the development of consistent treatment recommendations.

## Case Report

**Fig. 1:**
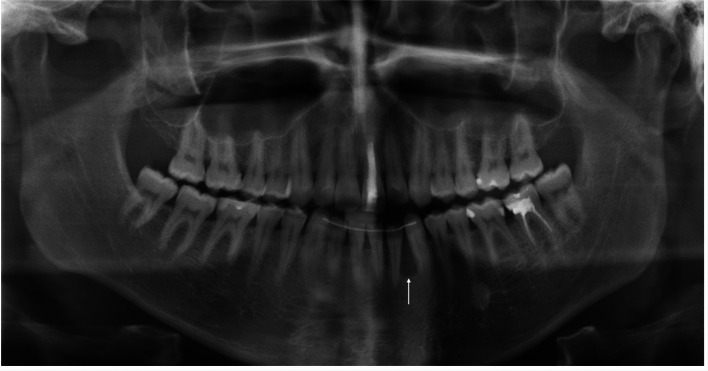
Initial radiological imaging: panoramic radiography exposed circumscribed radiolucency (size 8 × 6 × 5 mm) between 32 and 33 (arrow)

**Fig. 2:**
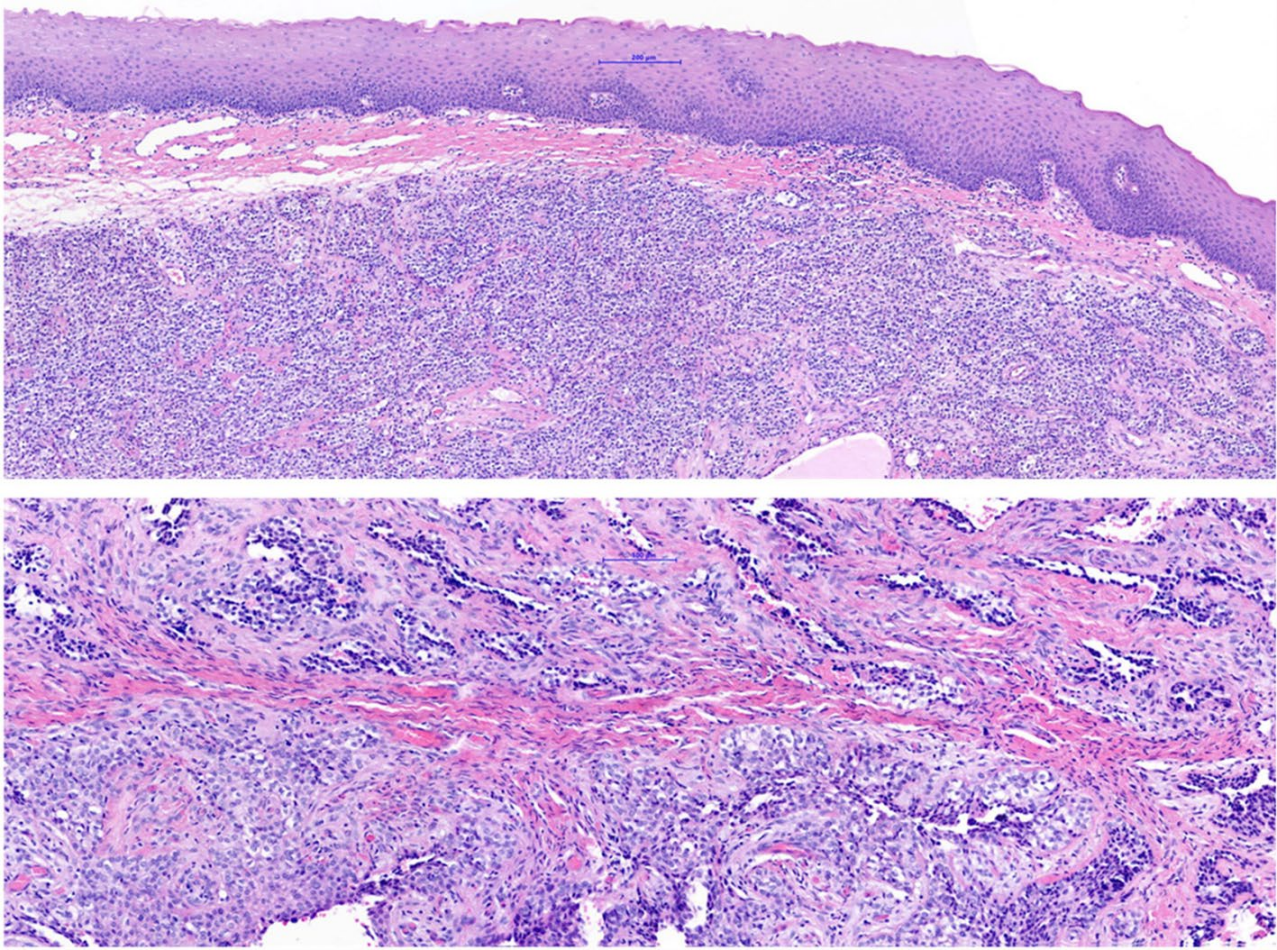
Histopathology: Hematoxylin–eosin staining (magnification × 100 in (**A**) and × 150 in (**B**)) of the biopsy sample revealed squamous cell mucosa infiltrated from below with the cells of clear cell odontogenic carcinoma, with striking clear cytoplasm. Higher magnification of clear cell odontogenic carcinoma shows sheets and trabeculae of monomorphic, polygonal cells with eosinophilic and clear cytoplasm

**Fig. 3:**
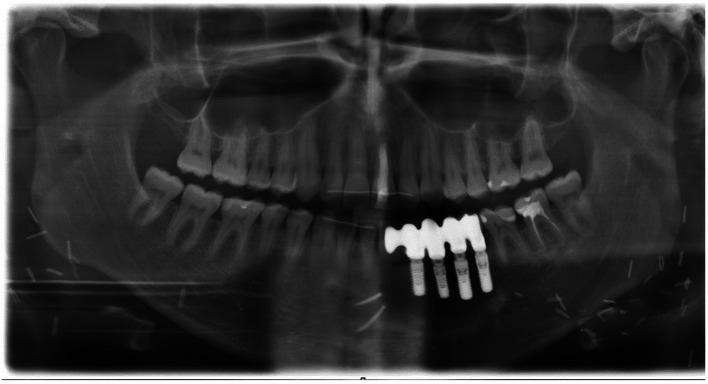
Postoperative radiological imaging: Panoramic radiography showed the final result after tumor resection and reconstruction via medial femoral condyle flap and implants in 32, 33, 34, and 35 with supraconstructions

A 25-year-old male patient was referred to the department of oral and maxillofacial surgery to evaluate a painless swelling of the left mandible that had persisted for about 6 months, and for further treatment of a cyst between 32 and 33. The patient’s medical history and physical examination were unremarkable. Panoramic radiography and the cone-beam computed tomography revealed a circumscribed radiolucency (size: 8 × 6 × 5 mm) between 32 and 33 (shown in Fig. 1). Under general anesthesia, a cystectomy of the left mandible was performed, and the sample was sent for pathological analysis. A histopathological diagnosis of CCOC was made. The microscopic examination of the sample revealed infiltration of the squamous mucosa with biphasic (solid and trabecular) tumor proliferation and focal perineural exophytic growth (Fig. 2). This proliferation consisted of unimorph cells with optically clear cytoplasm and slightly pleomorphic nuclei. Tumor cells showed no increased mitotic activity, a positive response to periodic acid–Schiff, and desmoplastic reactions. Immunohistochemically, the neoplastic cells showed a positive nuclear reaction to p63, and a Ki-67 score of less than 1%. Positron emission and computed tomography staging revealed no metastases. Due to the malignant behavior and the locally invasive growth of the CCOC, surgical intervention under general anesthesia was indicated. Marginal mandibulectomy and resection of the surrounding soft tissue were conducted from 35 to 41. Subsequently, a selective neck dissection was performed on the left side and a supraomohyoid neck dissection on the right side. The defect in the oral cavity was covered with a local flap reconstruction. Histopathologically, in-sano resection was confirmed with bland neck samples. The case was discussed in the interdisciplinary tumor board. Due to the young age of the patient and the positive postoperative result, adjuvant radiotherapy was dispensed with. After 5 months of inconspicuous clinical and radiographic follow-up, osseous reconstruction with a medial femoral condyle flap was implemented (shown in Fig. 3). Implants in positions 32, 33, 34, and 35 were performed 27 months later to restore oral functionality. In the continuous clinical and radiographic follow-up, currently 7.5 years after surgery, there were no signs of tumor recurrence.

## Discussion

CCOC mainly occurs in the 6th decade of life and predominantly affects women, in contrast to the case of a young man reported here [1, 5]. Consistent with our study, Labrador et al. observed that the mandible (82%) was concerned significantly more often than the maxilla (18%) [1]. In the present case, the patient’s only symptom was a painless swelling of the left mandible. Swelling or lumps (80%) were also the most common clinical symptoms in the literature, while pain occurred in only 41% of the 117 patients described. [1] Due to the nonspecific clinical and radiological features of CCOC, immunohistology is of particular importance. As initially, both benign (e.g., cystic lesions) and malignant processes (e.g., squamous cell carcinoma) are possible differential diagnoses. Before histopathological confirmation, a whole series of clear cell containing tumors must be considered as a differential diagnosis. Other odontogenic tumors (amyloid-rich odontogenic fibroma, calcifying epithelial odontogenic tumor, or odontogenic carcinoma with dentinoid), salivary gland tumors (clear cell carcinoma, mucoepidermoid carcinoma, or epithelial myoepithelial carcinoma), or distant metastases (renal clear cell carcinoma or malignant melanoma) must be ruled out. [6] In line with Ebert et al., therapeutic treatment of CCOC requires primary surgical tumor resection with adequate safety margins [5]. Depending on tumor staging, the necessity and extent of cervical lymph node dissection and adjuvant radiotherapy are determined. Following a one- or two-stage approach, reconstruction of bone and soft tissues completes the comprehensive interventional procedure. Continuous follow-up is crucial for the patient’s long-term outcome.

## Data Availability

The data that support the findings of this study are available from the corresponding author upon reasonable request.
